# Chronic Effects of Resistance Training in Breast Cancer Survivors

**DOI:** 10.1155/2017/8367803

**Published:** 2017-08-01

**Authors:** Wanderson Divino Nilo dos Santos, Paulo Gentil, Rafael Felipe de Moraes, João Batista Ferreira Júnior, Mário Hebling Campos, Claudio Andre Barbosa de Lira, Ruffo Freitas Júnior, Martim Bottaro, Carlos Alexandre Vieira

**Affiliations:** ^1^Federal University of Goiás (UFG), Goiania, GO, Brazil; ^2^Federal Institute of Education, Science and Technology of Southeast of Minas Gerais, Campus Rio Pomba, Rio Pomba, MG, Brazil; ^3^University of Brasília (UnB), Brasília, DF, Brazil

## Abstract

*Objective. *To analyse effects of resistance training (RT) in breast cancer survivors (BCS) and how protocols and acute variables were manipulated.* Methods.* Search was made at PubMed, Science Direct, and LILACS. All articles published between 2000 and 2016 were considered. Studies that met the following criteria were included: written in English, Spanish, or Portuguese; BCS who have undergone surgery, chemotherapy, and/or radiotherapy; additional RT only; analysis of muscle performance, body mass composition (BMC), psychosocial parameters, or blood biomarkers.* Results.* Ten studies were included. PEDro score ranged from 5 to 9. Rest interval and cadence were not reported. Two studies reported continuous training supervision. All reported improvements in muscle strength, most with low or moderate effect size (ES), but studies performed with high loads presented large ES. Five described no increased risk or exacerbation of lymphedema. Most studies that analysed BMC showed no relevant changes.* Conclusions.* RT has been shown to be safe for BCS, with no increased risk of lymphedema. The findings indicated that RT is efficient in increasing muscle strength; however, only one study observed significant changes in BMC. An exercise program should therefore consider the manipulation of acute and chronic variables of RT to obtain optimal results.

## 1. Introduction

The term “cancer” refers to a set of more than 100 diseases. Cancer is one of the leading causes of morbidity and mortality worldwide with an incidence of around 14.1 million cases and approximately 8.2 million deaths in 2012 [1]. Breast cancer is the most common form of cancer among women and in 2012 presented approximately 1.7 million cases worldwide [1]. Breast cancer aetiology is not fully understood, but it seems to have multifactorial causes involving reproductive and endocrine factors such as nulliparity, hormonal history, and the use of hormone therapy (contraceptive and hormone replacement). Other factors have also been associated with breast cancer, such as exposure to ionizing radiation, use of alcohol, high-calorie diets, physical inactivity, and obesity [1–3].

Breast cancer treatment includes surgery, chemotherapy, radiation, and hormone therapy, which can be used alone or in combination. Although aimed at a cure, cancer treatment has numerous deleterious side effects, diminishing patient quality of life. It has been reported in the literature that treatment can induce lymphedema [4–6], sedentary behaviour [7, 8], decreased aerobic fitness and muscle strength [9, 10], fatigue [11, 12], weight gain and changes in body composition [13], decrease in bone mineral density [14], high inflammatory profile [15, 16], immunosuppression [17, 18], peripheral neuropathy [19], changes in the perception of body image, anxiety, and depression [20–22]. These factors are commonly associated with treatment and can cause a downward spiral, reducing physical function and worsening the symptoms related to fatigue, which increases the risk of developing other diseases and reduces life expectancy in this population.

Regular exercise is becoming increasingly popular as an alternative treatment due to its ability to disrupt this downward spiral, minimise treatment side effects, and improve a survivor's quality of life [23]. Regular exercise has also shown physiological and psychological benefits, including positive changes in levels of fatigue and mood disorders (i.e., anxiety and depression) [24, 25]. Studies involving aerobic and resistance exercises have shown interesting effects in reducing fatigue levels, increasing functional capacity and muscle strength, and inducing positive changes in body composition and quality of life [24, 26–36]. Aerobic and resistance training protocols performed in combination (on different days) or concurrently (at the same session), however, have resulted in divergent outcomes in breast cancer survivors [32, 37–39]. Resistance training performed alone has also had contradictory effects on strength gain and changes in body composition in this population [40–43].

Paoli et al. [44] pointed out that in order to design a resistance training programme it is necessary to properly handle the acute variables related to training, such as muscle actions, type of resistance used, intensity (load), volume (total number of sets and reps), exercise selection, exercise order, rest intervals between sets, velocity (speed of execution), and training frequency [44, 45]. The different findings on strength gain and changes in body composition can be attributed in part to the different design of the training protocols [31, 32, 37–43, 46]. Current reviews of resistance training and cancer survivors have looked at safety and efficacy and at the effects of resistance training outcomes [47, 48]; however, to the best of the authors' knowledge, there is no systematic review that has aimed to critically analyse the acute training variables and how the resistance training protocols have been manipulated and designed in breast cancer survivors. This information will help researchers and health professionals to standardise and optimally design efficient resistance programmes in breast cancer survivors. The purpose of this systematic review is thus to analyse studies of the effects of resistance training in breast cancer survivors and how the resistance training protocols and the acute variables were manipulated in these studies.

## 2. Methods

### 2.1. Search Strategy

The current study follows the criteria of PRISMA* (Preferred Reporting Items for Systematic Reviews and Meta-Analyses)* in developing a systematic review [49].

Article searches were conducted by two researchers. The databases examined included: PubMed, Science Direct, and LILACS. Each researcher searched for articles individually, and after the searches, the researchers compared their findings and eliminated duplicated items.

The search terms were all possible combinations of the terms:* “weight training”*,* “strength training”*,* “resistance training”*,* “resistance exercise”, and “breast cancer” separated by the* “AND” operator (i.e., resistance training AND* breast cancer*). The search was conducted from February to April 2016 and the articles selected were published between 2000 and 2016.

### 2.2. Eligibility Criteria

All studies involving women breast cancer survivors who had undergone surgery, chemotherapy, and/or radiotherapy were included in the initial analysis. These studies should have objectively evaluated and/or applied an intervention with only resistance training (i.e., training with free weights, machines, and/or barbells). Only randomised clinical studies published in English, Spanish, or Portuguese were selected. The expected outcomes should involve at least one of the following variables: muscle performance involving objective measures of force (i.e., isokinetic strength, maximal strength (one-repetition maximum, 1 RM), multiple repetitions, and grip strength), body composition, psychosocial parameters (fatigue, depression, and quality of life), and blood biomarkers.

We excluded systematic review and/or meta-analyses, guidelines, letters to the editor, animal studies, studies in the paediatric population and other cancers (i.e., prostate cancer, lymphomas, etc.), studies using combined interventions (i.e., aerobic exercise and resistance training, among others) or nonconventional exercise prescription (e.g., aqua aerobics, Tai Chi, and yoga), studies that showed no objective measures of muscle performance, and studies that provided no consistent information regarding the experimental protocols used (i.e., type and/or number of exercises, sets, repetitions, training frequency, etc.). The reviewers had to be in agreement about the selection or exclusion of a study. In cases of disagreement, the opinion of a third reviewer was requested.

### 2.3. Data Extraction

The data extracted was authors, year of publication, description of the acute variables of resistance training protocol (volume, intensity, frequency, cadence, rest intervals, supervision ratio, and duration of the intervention), outcomes on muscular performance and body composition, sample characteristics, periods and types of evaluation, study results, and conclusions.

### 2.4. Methodological Quality and Strength of Evidence

The methodological quality of the studies in this systematic review was assessed by two independent reviewers using the Physiotherapy Evidence Database (PEDro) scale [50]. PEDro scale has been shown to have good levels of validity and reliability [50]. This scale evaluates the risk of bias and the statistical reporting of randomised controlled trials (RCTs) and is comprised of 11 items. The total PEDro score ranges from zero to 10 points, RCTs receiving less than six were considered to be of low quality (LQ), and those with a score six or greater were considered of high quality (HQ). The divergent scores were resolved by a third reviewer.

Effect size (ES) calculation was used to examine the magnitude of RT effect on BCS. Cohen's *d* ranges of 0.20, 0.50, and 0.80 were used to define small, medium, and large *d* values (*d* = ([*M*  pre − *M*  post]/SD  pooled)), respectively, calculated according to Cohen [51]. Values below 0.2 were classified as trivial.

In order to illustrate data, forest plots were done using the Review Manager Software (RevMan software package version 5.3) using the effect size (weighted mean difference, Hedges'* g*) and 95% confidence interval (CI) using a continuous random effects model for muscle strength and body composition.

## 3. Results

**Figure 1:**
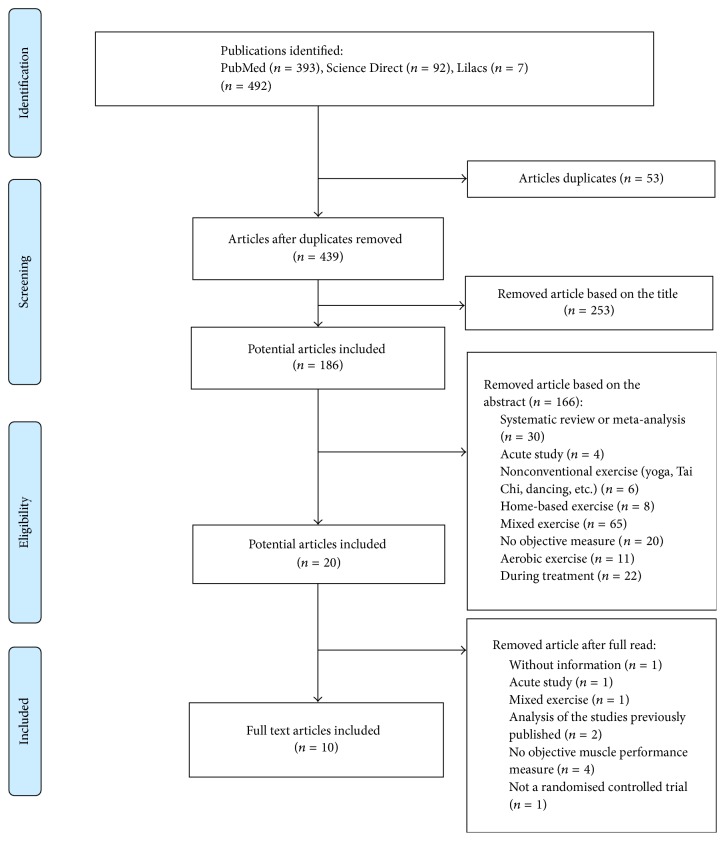
Flow diagram of selection process using PRISMA.

Between February and April 2016, 492 articles were identified for potential inclusion in the review. After an initial screening, 186 citations remained for further evaluation. Following the second screening, the remaining 20 potential articles were read and analysed. Finally, only 10 articles were selected for the review (Figure 1).

### 3.1. Methodological Quality of Studies

The methodological qualities of the studies are reported in Table 1. The median PEDro score for trials was 8 (range from 5 to 9). Nine trials were considered HQ and presented a low risk of bias [40–42, 52–57], and one study was considered LQ [40].

### 3.2. Description of Studies

All studies were published between 2005 and 2016. The sample size ranged from 39 to 295 participants. A total of 1448 women were evaluated, although 779 women participated in more than one study. Thus 669 women were effectively examined by the studies. The main outcomes are shown in Table 2.

Resistance training was performed twice a week in eight studies [40, 42, 43, 52, 54–57] and three times a week in another two studies [41, 53]. Exercise intensity for upper body ranged from low load, around 0.5 lb [43, 52, 54], to high load, 8 RM [41, 53]. The exercise load for lower body muscles was equivalent to 8–10 RM [43, 52, 54]. Training volume ranged from 2 to 3 sets and from 8 to 12 repetitions per exercise [40–43, 52–57]. Rest interval and movement velocity (speed of execution) were not reported in any study. There was continuous supervision only in 2 studies [41, 53]. Training periods ranged from 4 to 24 months [40–43, 52–57]. Additional information about the resistance training programmes is presented in Table 3. Resistance training significantly augmented muscle strength in all studies [40–43, 52–57]. Cohen's* d* effect size for muscle strength was medium to large, ranging from 0.59 to 1.10 [40, 42, 52, 53, 55, 56] and from 0.76 to 1.71 [40, 42, 52, 53, 55, 56] for upper and lower body muscles, respectively (Table 4). Experimental groups did not present increased risk or exacerbation of lymphedema symptoms [41, 42, 52, 53, 55]. Resistance training improved fatigue scores [53], quality of life [53, 54, 56], body image [56], psychosocial assessment [54], and bone mineral density [57].

In the studies reviewed, no significant changes were observed in BMI [40–43, 55], body weight [40, 42, 43, 55], lean body mass [40, 42, 55], body fat [40, 42, 43, 55], and waist circumference [43]. Most studies did not find changes in body fat percentage [40–42, 55]. Only one study found a significant increase in lean body mass and a reduction in body fat percentage [43]. Three studies reported a low effect size (*d* = −0.07 to −0.08) on body fat [40, 42, 55], and another one reported a large effect size (*d* = −0.85) [43] (Table 5).

**Figure 2:**
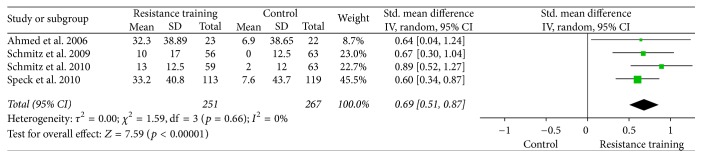
Forest plot on upper body strength (bench press: 1 RM, lb).

**Figure 3:**
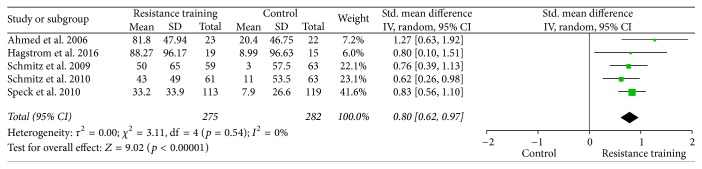
Forest plot on lower body strength (leg press: 1 RM, lb).

**Figure 4:**
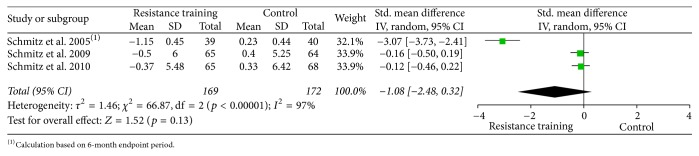
Forest plot on body composition: body fat (%).

**Figure 5:**
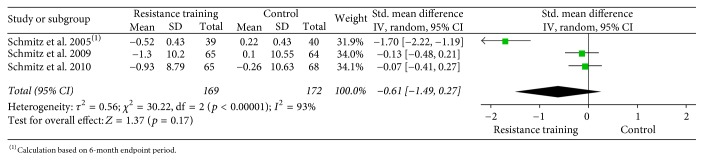
Forest plot on body composition: fat mass (kg).

**Figure 6:**
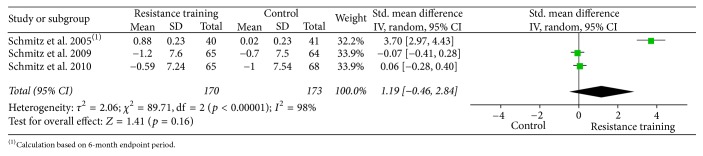
Forest plot on body composition: lean body mass (kg).

Forest plots for upper body strength, lower body strength, body fat percentage, fat mass, and lean body mass are presented from Figures 2–6.

## 4. Discussion

Resistance training is known to induce positive muscle adaptations, even in BCS [58]; however, there is no consensus or guidelines concerning the optimal design for resistance training programmes in order to induce greater muscle strength and alterations in body composition in this population. The aim of the present systematic review was thus to analyse the effects of resistance training in BCS and to analyse the resistance training protocols used in these studies. Ten studies were included in the review and, in accordance with the PEDro scale, nine were considered of high quality and one was considered of low quality. The findings showed that resistance training is efficient in increasing muscle strength in BCS; however, except for one study [43], it did not appear to alter body composition.

The resection of lymph nodes can change lymph flow and cause abnormal member oedema, which is classified as lymphedema. Previous studies to 1995 recommended avoiding repetitive or vigorous exercise for upper limbs because it could induce lymphedema [59, 60]. However from 2000, new researches demonstrate that repetitive and vigorous exercise as Dragon Boat Racing can be safe [61]. Sagen et al. [62] researched influence of physical activity on the development of arm lymphedema. Women who had axillary node dissection were separated into two different rehabilitation programs that lasted for 6 months: a group of no activity restrictions in daily living combined with a moderate resistance exercise program and another group with an activity restrictions (AR) program combined with a usual care program. No difference was found between groups, so little adverse effects were found between groups of no activity restrictions. However, a BMI > 25 was a risk factor for the development of lymphedema. More recently, Cormie et al. [63] showed that resistance exercise performed with both high- (6–8 RM) or low-load (15–20 RM) exercises for upper limbs caused no increased risk of lymphedema and was well tolerated by BCS. Notwithstanding, we did not find an increase in the appearance or exacerbation of oedema of the ipsilateral limb surgery with resistance training when compared to the control groups in any study reviewed. Nelson [64] observed that progressive resistance training did not increase the risk or severity of symptoms or even exacerbate lymphedema after a resistance training period ranging from 4 to 12 months. The studies in the present systematic review [41, 42, 52, 53, 55] are in agreement with these previous studies [63, 64], since no risk of lymphedema was found in any study.

An important issue for BCS is the control of body weight, because an increase in body weight above 10% is associated with increased mortality risk [65]. Women who have undergone chemotherapy have a 2.1 times greater risk of weight gain when compared to women without breast cancer [66]. Obesity can also double the risk of recurrence and death in breast cancer survivors [2]. Changes in body weight are the result of various factors, such as physical inactivity, decreased resting metabolic rate, excessive food intake, and hormonal changes [13]. Resistance training can therefore potentially have an important role in the control of body composition [67], but surprisingly, the current systematic review found only one study that observed significant changes in body composition [43]. Schmitz et al. [43] reported that resistance training resulted in a significant increase in lean body mass and a significant reduction in body fat. The body fat effect size was moderate (*d* = −0.52) for the control group (started training six months after the end of treatment) and large (*d* = −0.85) for the experimental group (started training immediately after the end of treatment). Schmitz et al. [55] and Brown et al. [40] did not report differences in body composition between the experimental and control groups; however, they reported lower body fat in the experimental group in comparison to the control group after the resistance training period, although there is a small effect size.

We further analysed the study protocols in order to understand the difference in the results reported. The methods used to evaluate body composition (DXA) and the training protocol adopted by Schmitz et al. [43] were very similar to Schmitz et al. [42] and Schmitz et al. [55]. The absolute fat loss after 12 months in Schmitz et al. [43], Schmitz et al. [42], and Schmitz et al. [55] was 1.47, 1.3, and 0.93 kg, respectively. The large effects size in Schmitz et al. [43] seems to be an artifact of the low standard deviation and there seems to be no clinically meaningful difference in fat loss among the studies. Based on this analysis, it does not seem plausible to suggest that resistance training promotes a clinically relevant reduction in body fat in BCS, nor is it possible to get insight into what RT protocol may be more suitable for that outcome. This lack of results seems to be related to training intensity, since the reviewed studies reported that participants increased the load based on subjective perceptions of discomfort [40], or after performing multiple sets with the same load for 2 to 4 consecutive training sessions [42, 43, 55]. When exercise is performed to or close to muscle failure, however, it is not possible to keep the number of repetitions constant in two consecutive sets while using the same load [68], which suggests that the participants were probably training at submaximal intensity. Considering that previous studies reported a significant loss in body fat as a result of resistance training usually involved high intensity protocols [69–72], the lack of adequate intensity may be the reason for the low reductions in body fat; however, it is important to test the feasibility of this type of training in BCS and at which stage it would be applicable. In addition to RT, aerobic training could potentiate changes in body composition [32, 38]; however these (aerobic exercise) effects were beyond the scope of the present review.

Another important factor for BCS is the maintenance and/or gain of muscle mass, since women with breast cancer who underwent chemotherapy showed a loss of muscle mass, mainly in the lower body [73], and the loss of lean mass can be worsened over time after treatment [74]. In this sense, RT is important both for maintenance and for increasing muscle mass in BCS, and it is an efficient tool to increase functional capacity and prevent sarcopenia and sarcopenic obesity [58]. We identified only one study that found a significant increase in lean mass [43] and large ES (ITG: *d* = 1.79 and DTG: *d* = 1.92), and two studies [42, 55] showed a reduction of lean mass in the EG at the end of 12 months as demonstrated by the negative ES (*d* = −0.16, *d* = −0.08, resp.).

The outcomes observed by Schmitz et al. [42, 43, 55] could be explained by basic different training protocols, and again intensity may have been the critical factor in the magnitude of the effect on muscle mass. In first study, [43] used more intense stimuli when working with loads close to maximal repetitions for lower limbs, whereas in others [42, 55] used a training programme with low progressive loads, without muscular failure, which may have resulted in differences between the studies.

Muscle strength is an important outcome, because higher levels of muscle strength are associated with lower mortality risk and a higher quality of life in different populations [75–81]. Six studies assessed the ES of upper body strength [40, 42, 52, 53, 55, 56]. The smallest ES was reported by Ahmed et al. [52], Brown et al. [40], Schmitz et al. [42], and Speck et al. [56], and the highest was seen in Brown et al. [40], Schmitz et al. [55], and Speck et al. [56]. When using the same protocol, the studies of Brown et al. [40] and Speck et al. [56] reported moderate ES in women with lymphedema and high ES in women without lymphedema; for that reason, the discrepancies among studies seem to be related to the presence of lymphedema. Considering that intensity was regulated by the subjective perception of discomfort in these studies, the use of lower intensities by patients with lymphedema may have led to the smaller ES seen in Brown et al. [40] and Speck et al. [56]. The same may be true for Ahmed et al. [52] and Schmitz et al. [42], which involved participants with lymphedema. Another study by Schmitz et al. [55] reported large ES in women without lymphedema when using the same protocol as Schmitz et al. [42]. The results therefore suggest that the different ES reported are due to the characteristics of the participants and suggest that the presence of lymphedema leads to a reduction in upper body strength gains. Psychological factors can affect performance during exercise; approximately 36% of patients with lymphedema report fear of using the affected limb, which induces less physical activity of the site and, consequently, a reduction of muscle strength when compared to the unaffected limb [82]. Such aspects may limit the magnitude of muscle strength gains for these women.

Six studies analysed the ES of lower body strength [40, 42, 52, 53, 55, 56]. Interestingly, patients with lymphedema generally reported smaller ES for lower body strength as well [40, 42, 56], and the analysis of patients without lymphedema had higher ES [40, 53, 55, 56]. The only exception was Ahmed et al. [52], which reported the highest ES for lower body strength (1.71) among the reviewed studies, in addition to involving participants with lymphedema. Once more, intensity may be the key. In Ahmed et al. [52], it was reported that the participants performed 8 to 10 RM and lifted the most weight they could in the lower body exercises, which suggests that training was performed with maximum loads.

As previously highlighted, an exercise programme should consider the manipulation of acute and chronic variables of resistance training in order to obtain optimal results [44, 45]. The present review found important weaknesses in training protocols; for example, many studies did not report the rest interval between sets, movement velocity, supervision ratio, and whether the exercise was performed until muscle failure. It is important to note that studies involving resistance training are usually limited to healthy people [83], which makes it difficult to design efficient and safe resistance training protocols for BCS. More detailed analyses of variable selection in BCS are needed.

A previous study in older people reported that shorter rest intervals (1 min) resulted in higher body composition and performance gains than longer rest intervals (4 min) [84]. On the other hand, McKendry et al. [85] demonstrated that one-minute rest interval may attenuate myofibrillar synthesis signalling compared to 5 minutes in young adults. The only known study to analyse resistance training variables in BCS was performed by Vieira et al. [86]. The authors investigated the acute effect of the rest interval between sets in women BCS [86]. They compared the effect of 1-minute versus 2-minute rest intervals on resistance training performance in BCS and women without breast cancer. The resistance training session was composed of three sets of 10 repetitions at 60° · s^−1^ of isokinetic unilateral knee extension. The results showed that peak torque and total work were significantly lower for the BCS group. The results also suggested that BCS may need rest intervals longer than 2 minutes to be able to fully recover; however, the chronic effects of recovery intervals on resistance training adaptations in BCS remain unknown.

The combination of the load used and muscle fatigue provided by resistance training, usually identified by repetitions leading to momentary muscle failure and falling performance in subsequent sets [68], may play an important role on resistance training adaptations [87, 88]. Protocols with a high load (3 sets with 85% of 1 RM), performed with rapid concentric contraction and 2 seconds for controlled eccentric phase without muscular failure, concentric and eccentric phases fast without muscular failure, and controlled concentric and eccentric actions (2 s for each muscle action) with muscle failure, were similar in strength gain and the hypertrophy of the elbow flexor muscles [89]. On the other hand, protocols with low loads (3-4 sets with 30–50% of 1 RM) may increase neuromuscular activation when repetitions are brought to momentary muscle failure [90, 91] and it has been demonstrated as able to promote strength gain and the muscular hypertrophy of the thighs [92] and arms [91]. Low-load resistance training performed to failure (3 sets with 30% of 1 RM) can lead to a similar increase in muscular strength and size in comparison to high-load training (1 set or 3 sets with 80% of 1 RM) [93]. Protocols with low loads (50% of 1 RM) and with controlled cycles of movements (3 s for concentric muscular contractions and 3 s for eccentric muscular contractions, without relaxation) were also able to elevate muscular strength and mass, similarly to high loads (80% of 1 RM), with rapid and intermittent movement cycles (1 s for concentric muscle contractions and 1 s for eccentric muscle contractions, 1 s pause) [94]. Note that these studies evaluated healthy subjects. According to the current review, studies that evaluated the effect of resistance training in BCS used low load for the upper body or high load for the lower body [43, 52, 54] and high load for the whole body [41, 53]. The training volume was from 2 to 3 sets and from 8 to 12 repetitions [40–43, 52–57]. Studies that used high loads and training volumes with 3 sets of 8 to 10 repetitions had a large effect size on lower limb [52, 53] and upper limb strength [53]; however, resistance training with progressive loads had a large and moderate effect size on BCS for lower and upper limb strength [40, 42, 52, 55, 56]. These outcomes are in agreement with studies that used low load, without reaching muscle failure, and that demonstrated an increase in the muscle strength but without an increase in the muscular mass [94, 95].

Muscle contraction velocity is also an important variable to be controlled, since it can alter the activation and production of power, presenting an important role in the improvement in functional capacity in the elderly [96]. Nogueira et al. [97] and Bottaro et al. [98] reported that older people performing RT at higher velocities showed greater gains in muscle size, strength, and functionality when compared with people that performed the same programme at lower velocities. Unfortunately, this variable was not reported in any of the reviewed studies, which precludes us knowing the potential effect in BCS.

Another variable that can affect the magnitude of resistance training adaptations is the training supervision ratio. Mazzetti et al. [99] examined the effect of resistance training with and without supervision and noted that the supervised group had higher muscular strength and fat-free mass gain when compared to nonsupervised group. Gentil and Bottaro [100] have found that a high supervision ratio (1 : 5 strength trainer to athlete ratio) induced higher strength gain in upper and lower body when compared to low supervision ratio (1 : 25) in young men. The present systematic review found that six studies reported a high training supervision ratio [41, 52–56], and only two studies reported continuous training supervision during the entire training period [41, 53]. Other studies mentioned that training sessions were supervised, but its ratio was not reported [40, 42]. Finally, studies with a high supervision ratio presented a large effect size for muscle strength gain [52, 53, 55, 56]; on the other hand, moderate effect size was observed in those studies in which supervision was not continuous [40, 42, 56]. Overall, these studies suggest that direct supervision during resistance training might be important for BCS.

## 5. Conclusions 

Resistance training seems to be safe for BCS, since it did not increase or exacerbate the risk of lymphedema. However, the effects of resistance exercise on BCS women in outcomes related to body weight and muscle strength appear to be higher, possibly due to the intensities adopted in the studies. An exercise programme should consider the manipulation of acute and chronic variables of resistance training in order to obtain optimal results. In this way, further studies should evaluate the effects of load, volume, rest intervals between sets, cadence (speed of execution), exercise choice and order, and training methods, on muscular adaptations in BCS so as to determine and consolidate the potential benefits of resistance training for this population.

## Figures and Tables

**Table 1:** Methodological quality and reporting of eligible studies PEDro scale.

First author, year	PEDro scale items^*∗∗*^											
	1	2	3	4	5	6	7	8	9	10	11	PEDro score (0–10)^*∗*^
Ahmed, 2006 [52]	Y	Y	N	Y	N	N	Y	N	Y	Y	Y	6
Brown, 2012 [40]	Y	Y	N	Y	N	N	N	N	Y	Y	Y	5
Hagstrom, 2016 [53]	Y	Y	Y	Y	Y	N	Y	Y	Y	Y	Y	9
Hagstrom, 2016 [41]	Y	Y	Y	Y	Y	N	Y	Y	Y	Y	Y	9
Ohira, 2006 [54]	Y	Y	Y	Y	N	N	Y	Y	Y	Y	Y	8
Schmitz, 2009 [42]	Y	Y	Y	Y	N	N	Y	Y	Y	Y	Y	8
Schmitz, 2005 [43]	Y	Y	Y	Y	N	N	Y	N	Y	Y	Y	7
Schmitz, 2010 [55]	Y	Y	Y	Y	N	N	Y	Y	Y	Y	Y	8
Speck, 2010 [56]	Y	Y	Y	Y	N	Y	Y	N	Y	Y	Y	8
Waltman, 2010 [57]	Y	Y	N	Y	N	N	N	Y	Y	Y	Y	6

N: no; Y: yes. ^*∗*^Scores of six or greater considered of high quality and scores of less than six considered of low quality. ^*∗∗*^PEDro scale items 1: eligibility criteria and source of participants; 2: random allocation; 3: concealed allocation; 4: baseline comparability; 5: blinded subjects; 6: blinded therapists; 7: blind assessors; 8: adequate follow-up; 9: intention-to-treat; 10: between-group comparisons; 11: point estimates and variability.

**Table 2:** Distribution of studies according to sampling, intervention, parameters, and main outcomes found.

Study	Sample	Group	Intervention length (months)	Parameters	Outcomes
Ahmed et al. [52]	*N* = 85 52 ± 7.7 years	EG and CG	6 months	Lower and upper body strength (1 RM) Lymphedema	EG increased muscle strength. Two subjects of CG and one of EG self-reported lymphedema; however there was no difference between groups (*p* = 4.0). Three women of CG reported lymphedema symptoms, while EG did not. Symptoms of lymphedema were not changed.

Brown et al. [40]	*N* = 295 EG = 56 ± 9 years CG = 57 ± 10 years	EG and CG	12 months	Body composition (DXA) Lower and upper body strength (1 RM)	EG had lower body fat than the CG after 12 months of intervention. However, no differences were found for other anthropometric parameters. EG improved muscle strength.

Hagstrom et al. [53]	*N* = 39 51.9 ± 8.8 years	EG and CG	4 months	Fatigue and quality of life by FACIT and FACT-G scales, respectively Godin Leisure-Time Exercise Questionnaire Lower (1 RM) and upper body strength (isometric)	Perceptions of fatigue and quality of life improved in EG compared to CG. EG improved muscle strength. Significant correlation between improvements in strength of the treated limb and improvements in global life quality in EG (*r* = 0.46, *p* = 0.004).

Hagstrom et al. [41]	*N* = 39 51.9 ± 8.8 years	EG and CG	4 months	Natural killer cell (NK) and natural killer T-cell (NKT) function and markers of inflammation (serum TNF-*α*, IL-6, IL-10, and CRP) Body composition Lower (1 RM) and upper body strength (isometric)	Lower NK and NKT cell expression of TNF-*α* in EG compared to CG. No change in body composition or in any inflammatory marker. EG improved muscle strength. Inverse correlations between changes in lower body strength and TNF-*α* expression on NK (*r* = −0.69, *p* = 0.001) and NKT cells (*r* = −0.36, *p* = 0.04). No adverse events, nor new cases of lymphedema.

Ohira et al. [54]	*N* = 86 EG: 53.3 ± 8.7 years CG: 52.8 ± 7.6 years	EG and CG	12 months	Body composition (DXA) Upper and lower boy strength (1 RM) Quality of life (CARES short form) Depressive symptoms (CES-D)	Physical global score increased 2.1% in TG and decreased 1.2% in CG. Psychosocial global score improved in EG (2.5%) compared to CG (0.3%). There were no changes in CES-D scores. Correlation between increases in upper body strength and improvements in physical global score (*r* = 0.32; *p* < 0.01) and psychosocial global score (*r* = 0.30; *p* < 0.01). Increases in lean mass correlated with improvements in physical global score (*r* = 0.23; *p* < 0.05) and psychosocial global score (*r* = 0.24; *p* < 0.05).

Schmitz et al. [43]	N = 85 52 ± 7.7 years	ITG and DTG	12 months	Body composition (DXA) Upper and lower body strength (1 RM) Plasma glucose and insulin, and hormones of IGF axis	ITG group increased lean mass and decreased body fat% compared to DTG from baseline to 6 months. Increase in upper and lower body muscle strength with training intervention. Reduction in IGF-II in both ITG and DTG groups. IGFBP-3 decreased in DTG group after 6 months of intervention.

Schmitz et al. [42]	*N* = 141 EG: 56 ± 9 years CG: 58 ± 10 years	EG and CG	12 months	Body composition (DXA) Upper and lower body strength (1 RM) Lymphedema	There were no differences in body composition between groups. EG had greater improvements in self-reported severity of lymphedema symptoms and muscle strength and a lower incidence of lymphedema exacerbations (14% versus 29%) in comparison to CG.

Schmitz et al. [55]	*N* = 134 EG 54 ± 8 years CG 56 ± 8 years	EG and CG	12 months	Body composition (DXA) Upper and lower body strength (1 RM) Lymphedema	Body fat% was lower in EG at 12 months. EG increased muscle strength. No between-group differences were observed in clinician-defined lymphedema onset or symptoms in secondary analysis limited to women with 5 or more nodes removed.

Speck et al. [56]	*N* = 295 56.5 years (36–80)	EG and CG	12 months	Body image and relationships scale (BIRS) Quality of life Upper and lower body strength (1 RM)	Greater improvement in BIRS total score in EG compared CG. EG improved self-perceptions of appearance, health, physical strength, sexuality, relationships, and social functioning. EG improved muscle strength.

Waltman et al. [57]	*N* = 249 58.69 ± 7.5 years	EG (also took medications) and CG (only took medications)	24 months	Bone mineral density and bone turnover (DXA) Hip and knee muscular strength (Biodex)	EG and CG improved body mineral density and bone turnover. EG had no additional improvements. EG improved muscle strength.

EG, experimental group. CG, control group. 1 RM, one-repetition maximum. DXA, dual-energy X-ray absorptiometry. CARES, cancer rehabilitation evaluation system. CES-D, center for epidemiologic studies depression scale. BCS, breast cancer survivors. ITG, immediate treatment group trained from months 0 to 12. DTG, delayed treatment group serving as control from 0 to 6 months and trained from months 7 to 12. FACIT, Functional Assessment of Chronic Illness Therapy. FACT-G, Functional Assessment of Cancer Therapy-General.

**Table 3:** Characteristics of resistance training protocols in the analysed studies.

Study	Exercises	Training load	Weekly frequency	Volume (sets × repetitions)	Rest interval	Session duration	Supervision ratio	Training progression
Ahmed et al. [52]	9 exercises involving arms, back, chest, buttocks, and legs.	Upper body exercises load starting at 0.5 lb, and 8–10 RM for lower body exercises	2x	3 × 8–10	—	~60 min	First 3 months at 1 : 4; then there was no supervision or it was 1 : 2.	—

Brown et al. [40]	Seated row, chest press, lateral or front raise, bicep curl, triceps pushdown, leg press, back extension, leg extension, and leg curl.	—	2x	2-3 × 10	—	90 min	First 3 months supervised, followed by 9 months with no supervision.	Exercise load was slowly increased if there were no lymphedema symptoms.

Hagstrom et al. [53]	Programme 1: leg extension, leg curl or Romanian deadlift, lat. pull down, machine bench press, seated row, back extension, prone hold, or sit ups. Programme 2: barbell squat, deadlift, free weight barbell bench press, leg press, bent over barbell row, and assisted chin up	8 RM	3x	3 × 8–10	—	60 min	1 : 1 or 1 : 2–5.	Load was increased when subjects performed 10 RM.

Hagstrom et al. [41]	Programme 1: leg extension, leg curl or Romanian deadlift, lat. pull down, machine bench press, seated row, back extension, prone hold, or sit-ups. Programme 2: barbell squat, deadlift, free weight barbell bench press, leg press, barbell bent over row, and assisted chin up.	8 RM	3x	3 × 8–10	—	60 min	1 : 1 or 1 : 2–5.	Exercise load was increased when subjects performed 10 RM.

Ohira et al. [54]	9 exercises involving chest, back, shoulders, arms, buttocks, hips, and thighs.	According to Schmitz 2005	According to Schmitz 2005	According to Schmitz 2005	According to Schmitz 2005	According to Schmitz 2005	First 3 months at 1 : 4; then there was no supervision or it was 1 : 2.	According to Schmitz 2005

Schmitz et al. [42]	Seated row, supine dumbbell press, lateral or front raises, biceps curl, and triceps pushdown, leg press, back extension, leg extension, and leg curl.	—	2x	3 × 10	—	90 min	13 weeks in small groups, followed by no supervision.	Exercise load was slowly increased when subjects completed 2 training sessions with no change in arm symptoms.

Schmitz et al. [43]	9 exercises involving chest, back, shoulders, arms, buttocks, hips, and thighs.	Upper body exercises load starting with no weight or at 0.5 lb and 8–10 RM for lower body exercises	2x	3 × 8–10	—	~60 min	13 weeks at small groups, followed by no supervision.	Upper body load: progressed as symptoms allowed. Lower body: weight was increased if subjects could perform 10 repetitions at each two sessions for the first 3 months. For the remaining months, participants increased the weight after four sessions during which they lifted the same weight for 10, 10, and 12 repetitions in each set.

Schmitz et al. [55]	Seated row, supine dumbbell press, lateral or front raises, biceps curl, and triceps pushdown, leg press, back extension, leg extension, and leg curl.	—	2x	3 × 10	—	90 min	13 weeks at 1 : 2–6, followed by no supervision.	Exercise load was slowly increased when subjects completed 2 training sessions with no change in arm symptom.

Speck et al. [56]	Seated row, supine dumbbell press, lateral or front raises, bicep curls, and triceps pushdowns, leg press, back extension, leg extension, and leg curl.	—	2x	3 × 10	—	90 min	13 weeks at 1 : 2–6, followed by no supervision.	Exercise load was slowly increased when subjects completed 2 training sessions with no change in arm symptom.

Waltman, et al. [57]	Biceps curl, overhead triceps or press and upward row, back and knee extension, side hip raise, and hip flexion and extension.	—	2x	2 × 8–12	—	—	Strength training took place in subject homes using free weights the first 9 months of the study, and at fitness centres the last 15 months.	Potential goals for progressive training were increases in weights of 20% the first 3 months of exercises, 10% at 6 and 9 months, 5% at 12, 15, and 18 months, and 3% at 21 and 24 months.

^*∗*^RM, repetition maximum.

**Table 4:** Muscle strength gain *d* effect size.

Studies	Condition	RT (ES *d*)	ES magnitude	Control (ES *d*)	ES magnitude
		Lower body strength^*∗*^			
Ahmed et al. [52]	—	1,71	Large	0,44	Small
Brown et al. [40]	Lymphedema	0,77	Medium	0,05	Trivial
Brown et al. [40]	Nonlymphedema	0,88	Large	0,21	Small
Hagstrom et al. [53]	—	0,92	Large	0,09	Trivial
Schmitz et al. [42]	—	0,77	Medium	0,05	Trivial
Schmitz et al. [55]	—	0,88	Large	0,21	Small
Speck et al. [56]	Lymphedema	0,76	Medium	0,02	Trivial
Speck et al. [56]	Nonlymphedema	1,00	Large	0,25	Small

		Upper body strength^*∗∗*^			
Ahmed et al. [52]	—	0,69	Medium	0,15	Trivial
Brown et al. [40]	Lymphedema	0,59	Medium	0,00	Trivial
Brown et al. [40]	Nonlymphedema	1,04	Large	0,17	Trivial
Hagstrom et al. [53]	Treated arm^*∗∗∗*^	0,88	Large	−0,13	Trivial
Hagstrom et al. [53]	Nontreated arm^*∗∗∗*^	0,95	Large	−1,11	Large
Schmitz et al. [42]	—	0,59	Medium	0,00	Trivial
Schmitz et al. [55]	—	1,04	Large	0,17	Trivial
Speck et al. [56]	Lymphedema	0,58	Medium	−0,01	Trivial
Speck et al. [56]	Nonlymphedema	1,10	Large	0,27	Small

RT: resistance training; ES: effect size. ^*∗*^Leg press (1 RM). ^*∗∗*^Bench press (1 RM). ^*∗∗∗*^Unilateral isometric chest press.

**Table 5:** Body composition *d* effect size.

Studies	Condition	RT (ES *d*)	ES magnitude	Control (ES *d*)	ES magnitude
		Body fat (%)			
Brown et al. [40]	Lymphedema	−0,08	Trivial	0,08	Trivial
Brown et al. [40]	Nonlymphedema	−0,07	Trivial	0,05	Trivial
Schmitz et al. [43]	ITG^*∗*^	−0,87	Large	0,19	Trivial
Schmitz et al. [43]	ITG versus DTG^*∗∗*^	−1,70	Large	−1,42	Large
Schmitz et al. [42]	—	−0,08	Trivial	0,08	Trivial
Schmitz et al. [55]	—	−0,07	Trivial	0,05	Trivial

		Fat mass (kg)			
Schmitz et al. [43]	ITG^*∗*^	−0,30	Small	0,13	Trivial
Schmitz et al. [43]	ITG versus DTG^*∗∗*^	−0,85	Large	−0,52	Medium
Schmitz et al. [42]	—	−0,13	Trivial	0,01	Trivial
Schmitz et al. [55]	—	−0,11	Trivial	−0,02	Trivial

		Lean body mass (kg)			
Schmitz et al. [43]	ITG^*∗*^	1,14	Large	0,03	Trivial
Schmitz et al. [43]	ITG versus DTG^*∗∗*^	1,79	Large	1,92	Large
Schmitz et al. [42]	—	−0,16	Trivial	−0,09	Trivial
Schmitz et al. [55]	—	−0,08	Trivial	−0,13	Trivial

^*∗*^Calculation based on 12-month endpoint. ^*∗∗*^Calculation based on 6-month period. ITG, immediate treatment group trained from months 0 to 12. DTG, delayed treatment group serving as control from 0 to 6 months and trained from months 6 to 12.
